# Do bicortical diaphyseal array pins create the risk of periprosthetic fracture in robotic-assisted knee arthroplasties?

**DOI:** 10.1186/s42836-021-00082-8

**Published:** 2021-07-05

**Authors:** Andrew G. Yun, Marilena Qutami, Kory B. Dylan Pasko

**Affiliations:** 1 Orthopedic Surgery, Center for Hip and Knee Replacement, Providence Saint John’s Health Center, 2121 Santa Monica Blvd, 90404 Santa Monica, CA USA; 2 Orthopedic Surgery, MedStar Georgetown University Hospital, 3800 Reservoir Rd NW, 20007 Washington, DC USA

**Keywords:** Knee arthroplasty, Shaft fracture, Complication, Pin site, Robotic-assisted arm

## Abstract

**Background:**

Optical array placement for robotic-assisted knee replacement introduces the rare, but real risk of periprosthetic fracture. The purpose of this retrospective study was to review the incidence of fracture with the conventional technique of bicortical diaphyseal pin placement. We also evaluated a modified method of unicortical periarticular pin placement to mitigate this risk.

**Methods:**

We reviewed 2603 knee arthroplasties that were performed between June 2017 and December 2019. The conventional bicortical diaphyseal technique was used in 1571 knees (bicortical diaphyseal group) and the unicortical periarticular technique was used in 1032 knees (unicortical periarticular group).

**Results:**

A more than 1-year follow-up revealed that 3 femoral shaft fractures (0.19%) occurred in the bicortical diaphyseal group and no fracture took place in the unicortical periarticular group. There was no array loosening in either group.

**Conclusions:**

The modified unicortical periarticular pin placement is a reliable technique for computer-navigated and robotic-assisted knee arthroplasties. It may be associated with a lower incidence of postoperative femoral shaft fractures, compared to conventional bicortical diaphyseal pinning.

## Introduction

Computer navigated and robotic-assisted techniques for total knee arthroplasty (TKA) and unicompartmental knee arthroplasty (UKA) require placement of temporary tracking pins for bone registration. While there is substantial literature supporting the benefits of these advanced techniques compared to conventional UKA and TKA, there is also the intrinsic associated risk of pin site complications [1–3].

Femoral or tibial shaft fractures at the pin site are severe complications that require emergent surgical intervention and may adversely affect the long-term outcomes. The minor pin-site complications, such as bleeding, suture abscess, and neuropraxia can be managed conservatively [4, 5]. Currently, the bicortical diaphseal (BD) technique is commonly used in computer-navigate and robotic-assited knee arthroplasties. The reported incidences of shaft fractures caused by array pinning range from 0.16 to 1.3% [6, 7].

The aim of this study was to report a novel technique of dual-pin placement using a unicortical periarticular (UP) technique for both the femur and tibia to mitigate the risk of fracture. We also retrospectively reviewed 2603 knee arthroplasties using the conventional BD technique and modified UP technique.

## Materials and methods

Between June 2017 and December 2019, a total of 2603 knee arthroplasties (1702 TKAs and 901 UKAs) were performed using a robotic-assisted arm (RAA) at a single institution and with a minimum of 1-year follow-up. The surgeries were performed by three senior surgeons using the Stryker Mako Robotic Assisted System (Stryker Kalamazoo, MI, USA). The Triathlon system was used for TKA, and the Mako Restoris system was used for UKA (Stryker Kalamazoo, MI, USA). The charts were retrospectively reviewed for complications, reoperation, and rehospitalization. The primary outcome measures were periprosthetic fracture and the need for reoperation. The secondary outcome measure was array loosening requiring discontinuation of the robotic-assisted procedure. The study was approved by the Institutional Review Board of the hospital.

The RAA manufacturer suggests that two BD pins be used for each optical array. The three surgeons initially followed the manufacturer’s guidelines for the BD technique. After three femoral fractures occurred, two surgeons switched to the UP technique described below in January 2019.

### Bicortical Diaphyseal Technique

As described and directed by the manufacturer, we used two Schanz pins to withstand the vibrational force of the robotic saw. We inserted two percutaneous Schanz pins (4.0 mm) into the diaphysis of the femur. The insertion points were located superior to the main incision, and the pins were inserted from anterior to posterior to obtain bicortial purchase. We inserted another two percutaneous Schanz pins into the diaphysis of the tibia. The insertion points were located distal to the main incision, and the pins were inserted from the antero-medial crest to the posterolateral cortex. Ancillary pin stabilizers were placed over the pins to increase the rigidity of the array fixation. A 2-dimensional locking bracket was used to secure each optical tracking array to the dual pin construct. The percutaneous wounds were closed with 3-0 Monocryl suture (Ethicon, Inc., Cornelia, GA, USA).

### Modified Unicortical Periarticular Technique

**Fig. 1:**
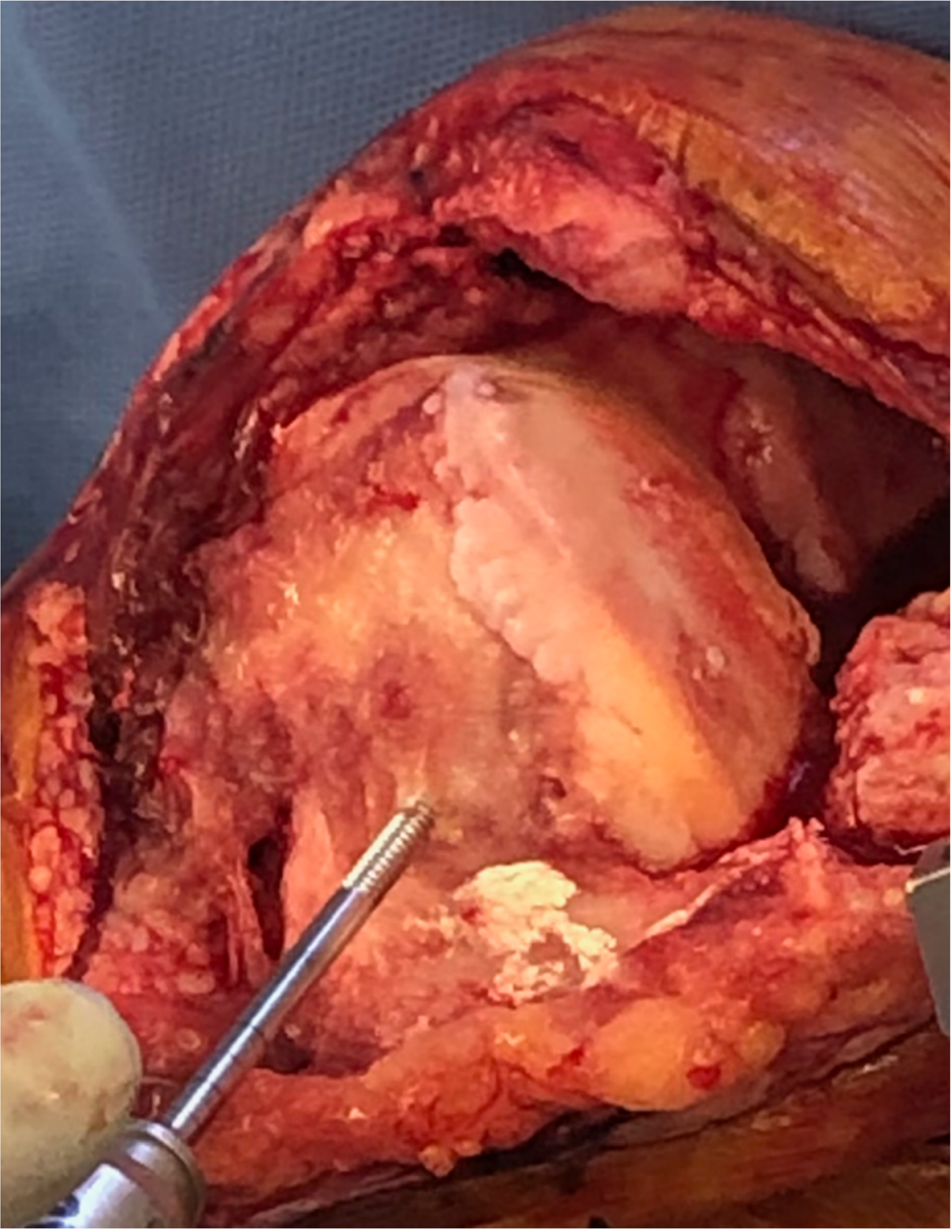
A unicortical femoral Schanz pin is inserted just anterior to the medial epicondyle

**Fig. 2:**
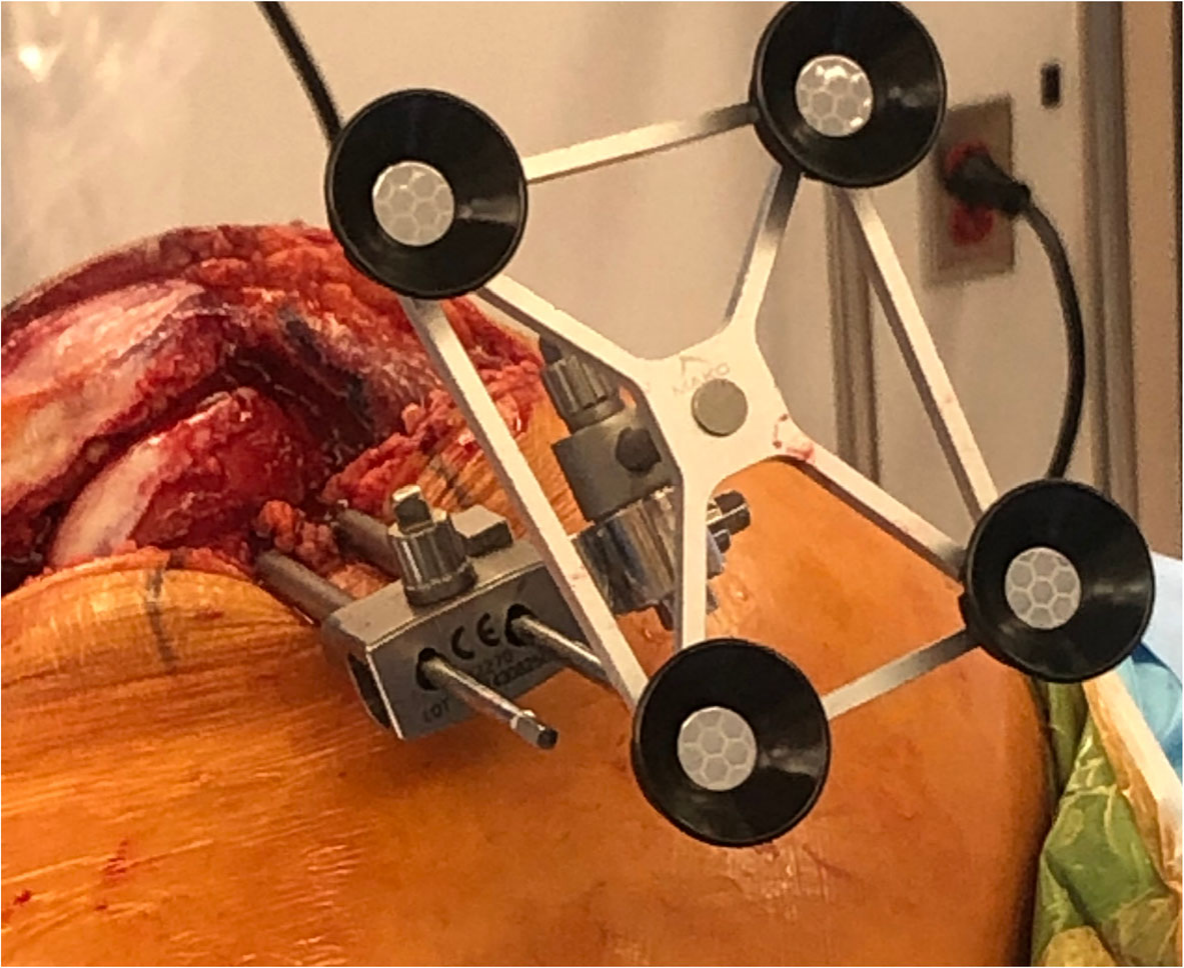
A modified 3-dimensional locking bracket is used to position the optical array in any position

**Fig. 3:**
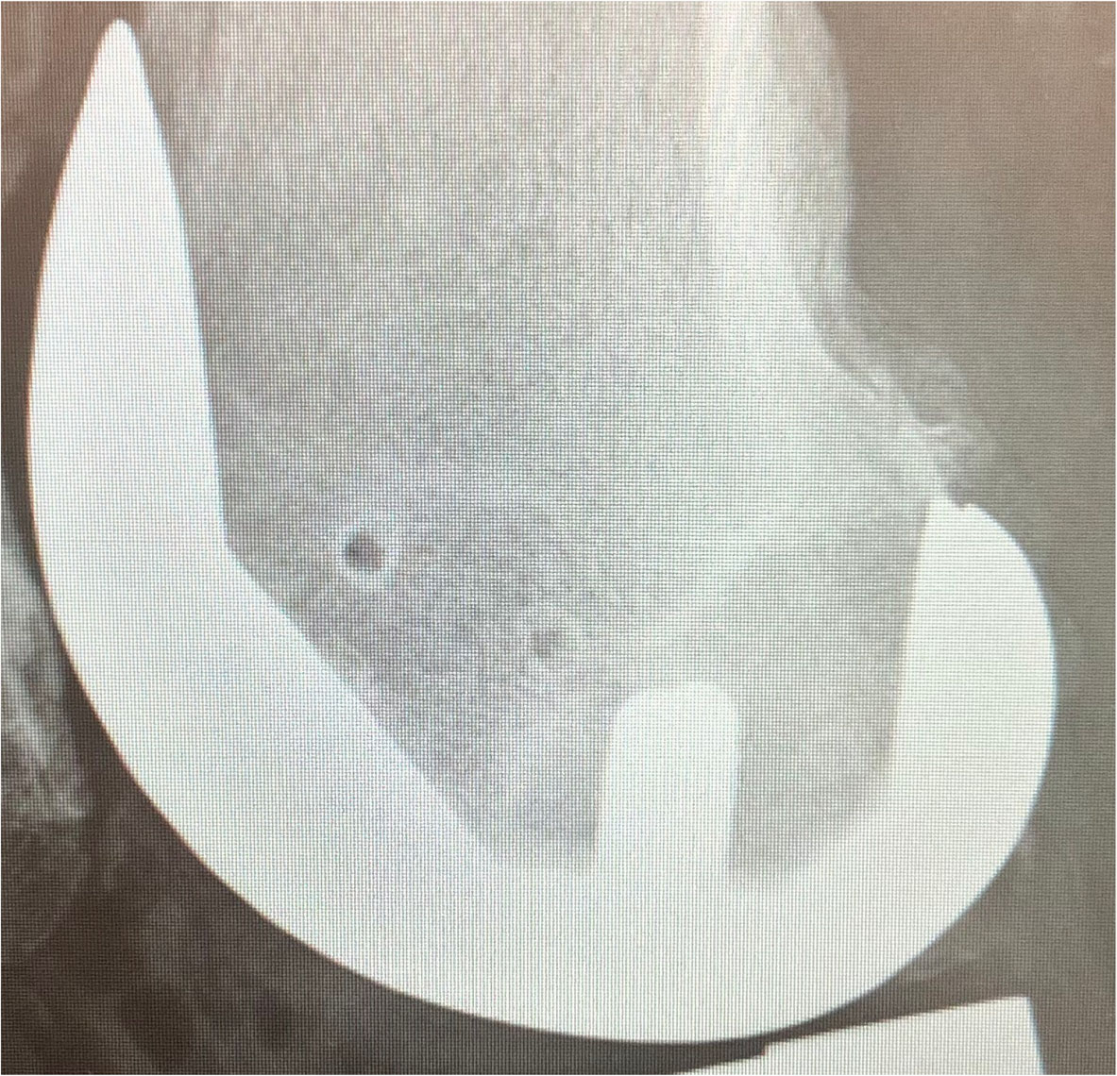
A lateral X-ray of TKA shows the position of the pin hole after periarticular femoral pin placement

We inserted two Schanz pins (4.0 mm) into the medial epicondyle of the femur through the main incision. As described by Owens et al. [4], the first insertion point was located 2 to 3 cm above the knee joint. The Schanz pin was inserted just anterior to the medial epicondyle. The pin was directed from medial to lateral and secured in the cancellous bone to a depth of 4 cm in a unicortical fashion (Fig. 1). A pin guide was placed over the first Schanz pin to facilitate the placement of a second Schanz pin in the same direction, and anterior and superior to the first pin. Care was taken to avoid transcortical drilling. A modified 3-dimensional locking bracket was used to position the optical array (Fig. 2). A postoperative lateral radiograph shows the modified pin placement (Fig. 3).

**Fig. 4:**
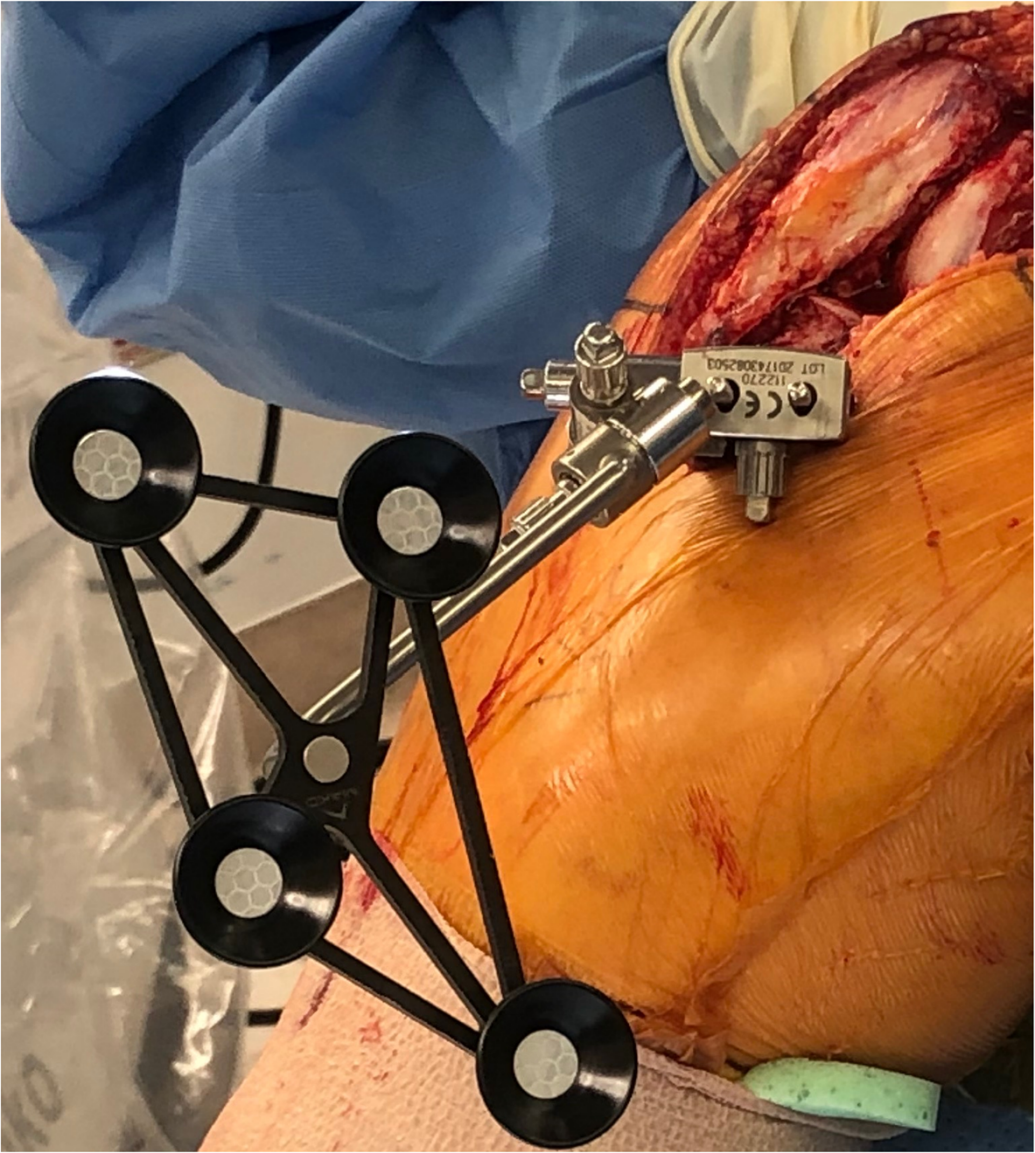
The tibial optical array is displaced distally from the pins with a 3-dimensional bracket to avoid impingement with the robotic saw

For the tibia, two Schanz pins were placed through the main incision. The first insertion point was located 2 cm inferior to the antero-medial plateau. The pin was directed to the posterolateral aspect of the tibia in the axial plane and horizontally in the coronal plane. The pin was advanced to a depth of 3 to 4 cm, and care was exercised to avoid penetrating the far cortex, because an injury to the neurovascular structures was possible. A pin guide was placed over the first Schanz pin, and a second Schanz pin was inserted below in the same direction. In order to avoid robotic saw impingement, a modified optical array with extended displacement (borrowed from the total hip array set) was used to shift the optical tracker distally by 10 cm. Another 3-dimensional locking bracket was employed to provide universal freedom for positioning the optical tracker (Fig. 4). The wounds were closed in the usual manner.

## Results

**Fig. 5:**
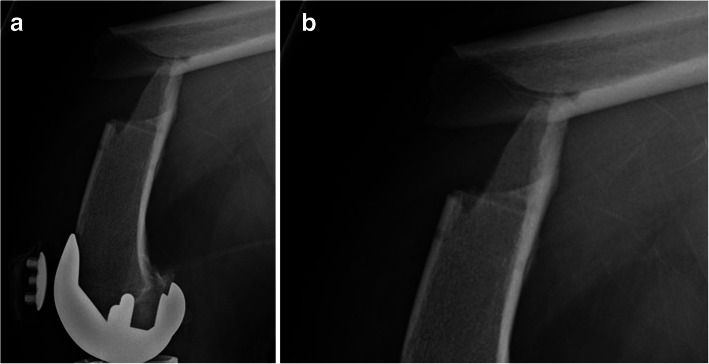
A. A lateral X-ray shows a short oblique periprosthetic femoral fracture. B. A close-up view shows the fracture originated from the stress riser left by the diaphyseal pin hole

**Fig. 6:**
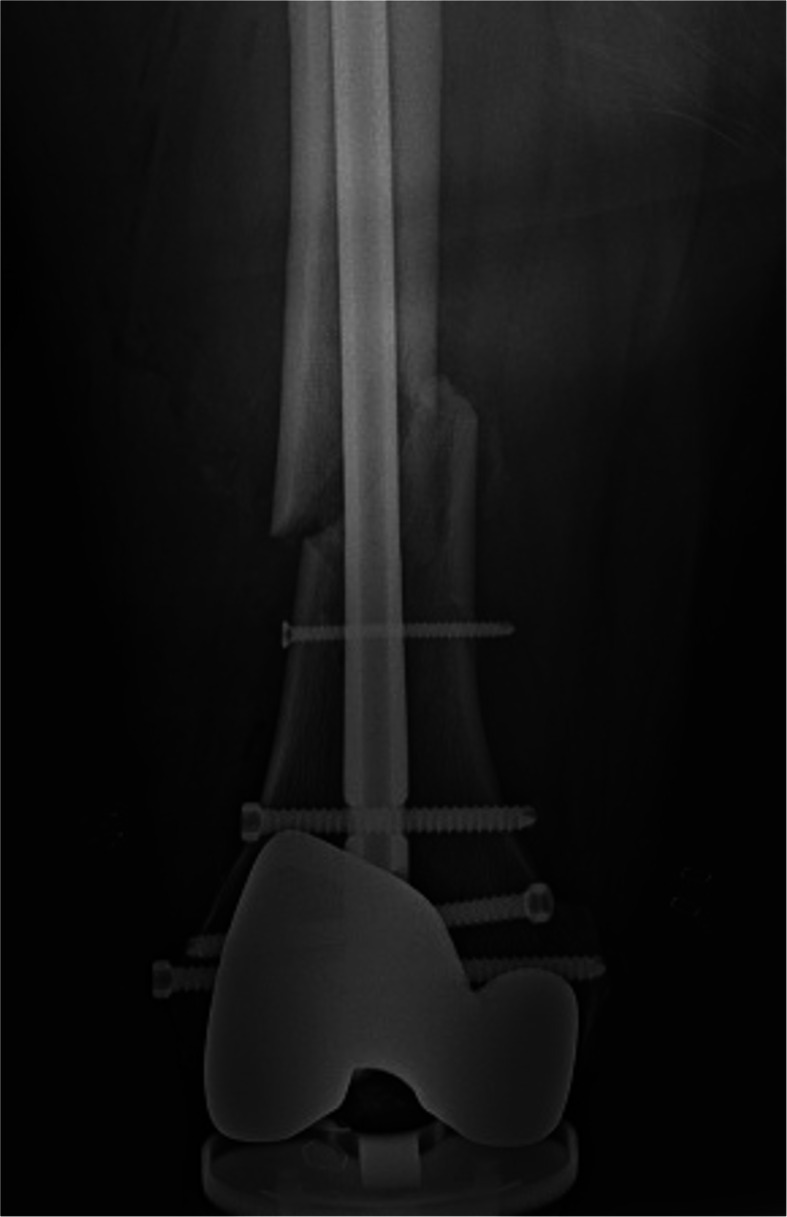
An anteorposterior X-ray shows the fracture is fixed with intramedullary femoral rodding

In a total of 2603 knees, the BD technique was used in 1571 (60%) knees (BD group), and the UP technique was used in 1032 (40%) knees (UP group). In the BD group, femoral shaft fractures occurred in 3 of 1571 (0.19%) limbs within the first 3 months after surgery and were caused by minor or no visible trauma. All fractures were closed injuries, and were of simple and oblique pattern. The fracture sites lied at the femoral array pin hole (Fig. 5A, B). Component loosening or instability was not observed. The fractures were treated with intramedullary femoral rodding (Fig. 6). The age, gender, body mass index (BMI), and diagnosis of osteoporosis of the 3 patients are described in Table 1. Conversely, no fracture was reported in the UP group. No array loosening was observed in either group.

**Table 1:** Age, gender, body mass index (BMI), and diagnosis of osteoporosis in femoral shaft fracture patients

Patient 1	
Age (yrs)	63
Gender	Female
BMI (kg/m^2^)	27
Osteoporosis	No
Patient 2	
Age (yrs)	57
Gender	Female
BMI (kg/m^2^)	45
Osteoporosis	No
Patient 3	
Age (yrs)	81
Gender	Female
BMI (kg/m^2^)	20
Osteoporosis	Yes

## Discussion

In robotic-assisted knee arthroplasties, the use of optical arrays has the advantages of achieving a better alignment and implant position, compared to the conventional techniques. However, a femoral or tibial shaft fracture due to the mechanical weakness caused by the pin holes is a catastrophic complication. The stress risers and ensuing mechanical weakness created by two bicortical pin holes may be an acceptable risk for those who support the benefits of RAA surgery. However, an alternative optical array placement method that facilitates the procedure without weakening the bone to the same degree may be far more preferable. It appears that even well-placed pin holes in the diaphysis intrinsically weaken the mechanical strength of the bone.

The complication of diaphyseal fracture arising from optical array pin holes in the shaft was previously identified with the advent of computer navigated TKA [7–9]. Beldame et al., [7] on the basis of a single surgeon’s experience, reported the incidence of fracture was as high as 1.3% in 385 knees. In a broader meta-analysis, Brown et al. [6] found the incidence of fractures through the pin site was 0.16% in either the femur or tibia. The recognized patient risk factors of fracture include obesity and osteoporosis, and the surgical factors identified included the placement of transcortical pins, wider diameter pins, and multiple drill holes [7, 9–11]. It is likely that a combination of these risk factors led to shaft fracture in these three patients.

There are three main technical concerns with bicortical pins in the diaphysis. Firstly, the location of the pin holes may influence the rate of fracture, and the diaphysis may be at higher risk than the periarticular bone. Beldame et al. warned that almost all postoperative fractures in his series originated at the diaphyseal pin hole [7]. Bonutti et al. reported 2 diaphyseal fractures before switching to metaphyseal pins [12]. Even with the conservative choice of unicortical pins, Blue et al. reported a displaced femoral shaft fracture 6 weeks after RAA TKA [13]. In the tibia, Hoke et al. reported 3 of 220 (0.13%) tibial shaft fractures, and also switched to pins in the metaphysis [11]. Based on our series, we agree that the periarticular bone may be more robust to torsional and bending stresses than the diaphysis.

Secondly, the manufacturer currently recommends percutaneous pins. Percutaneous pins, however, are difficult to place completely centered on bone in obese or muscular thighs. While unintentional, it is not uncommon to have drilled an eccentric-transcortical pin. The error of eccentric drilling becomes clear only on the postoperative radiograph. Jung et al. graphically articulated the increased risk of fracture caused by transcortical pins [10]. Clinically, Beldame et al. reported all 5 fractures originated from transcortical pins [7]. In our experience, placing the pins under direct vision within the incision not only eliminated the risk of transcortical drilling, but also seemed far easier.

Thirdly, placing a pin through both cortices in the diaphysis weakens the bone. Bonnutti et al. calculated that bicortical pin holes double the bone weakness compared to unicortical pins [12]. In contrast, Owens and Swank reported no fractures in 984 knees using a unicortical method [4]. We also found the two unicortical pins near the metaphysis to be sufficient to maintain array stability, even in osteoporotic bone. Each pin in the femur had secure purchase with at least 4 cm of cancellous bone and the ancillary pin stabilizers further increased the array fixation. Despite the concerns of greater vibrational forces from the RAA saw, we had no cases of array loosening. Furthermore, the femoral array pins are positioned 1 to 2 cm proximal to the femoral bone cuts. The placement of these pins away from the proposed saw cuts can be confirmed virtually during the planning stages.

As a technical hint, the femoral pins should be directed horizontally to avoid an injury to the nearby neurovascular structures [14]. In addition, we learned that placing the pin holes within 2 to 3 cm proximal to the joint line requires a modification of the optical arrays and connection systems. The femoral array needs an additional junctional bracket to provide the correct angle for optical visualization (Fig. 2). The optical portion of the tibial array should be displaced distally from the pins so that it does not impinge on the saw. With these modifications, we are able to successfully proceed with registration and bone preparation.

This study has several limitations. A primary concern is the short follow-up time. Although most series reported fractures occurring within the first 3 months, there are also reports of a delayed femoral fracture through a pin site [7, 8]. Therefore, it is possible that the incidence of pin site fracture may increase with time. Furthermore, the decrease in fractures in the UP group may have been due, in part, to the learning curve, as the two surgeons who switched from the BD technique may have become more skilled with pin placement over time. Another major limitation is the absence of a comparison of bone density between the two groups since osteoporosis is a known risk factor for fracture in this population. Moreover, this study is limited by the absence of radiographic assessment of component alignment in two planes between the two groups. Although a change in pin placement would theoretically affect only bone registration and optical tracking, a future comparative study of radiographic alignment is warranted. Ideally, demographically comparable groups could be analyzed for any alteration in accuracy with a modified pin placement. The study is also limited by our choice to defer determination of statistical significance in the varying incidence of fractures between the two groups due to the rarity of the event itself. A final concern is that the modified UP technique is currently off-label.

## Conclusions

The modified UP technique is reliable for computer-navigated and robotic-assisted knee arthroplasties. It may be associated with a lower incidence of postoperative femoral shaft fracture originating from the array pin holes.

## Data Availability

Supporting data are available upon request.

## Abbreviations

BD: Bicortical diaphyseal
UP: Unicortical periarticular
TKA: Total knee arthroplasty
UKA: Unicompartmental knee arthroplasty
RAA: Robotic assisted arm
BMI: Body mass index
